# Hybrid Approach in Acute and Chronic Aortic Disease

**DOI:** 10.3390/medicina58010049

**Published:** 2021-12-29

**Authors:** Michele Murzi, Pier Andrea Farneti, Antonio Rizza, Silvia Di Sibio, Cataldo Palmieri, Marco Solinas

**Affiliations:** 1 Adult Cardiac Surgery Division, Geatano Pasquinucci Heart Hospital, Fondazione Toscana Gabriele Monasterio, 54100 Massa, Italy; farneti@ftgm.it (P.A.F.); disibio@ftgm.it (S.D.S.); solinas@ftgm.it (M.S.); 2 Interventional Cardiology Division, Geatano Pasquinucci Heart Hospital, Fondazione Toscana Gabriele Monasterio, 54100 Massa, Italy; rizza@ftgm.it (A.R.); palmieri@ftgm.it (C.P.)

**Keywords:** aortic aneurysm, endovascular therapy, aortic arch

## Abstract

The management of patients with aortic disease that involves the ascending aorta, the aortic arch, and the descending aorta represent a surgical challenge. Open surgical repair remains the gold standard for aortic arch pathologies. However, this operation requires a cardiopulmonary bypass and a period of profound hypothermia and circulatory arrest, which carries a substantial rate of mortality and morbidity. For these reasons, hybrid arch repair that involves a combination of open surgery with endovascular aortic stent graft placement has been introduced as a therapeutic alternative for those patients deemed unfit for open surgical procedures. Hybrid repair requires varying degrees of invasiveness and can be performed as a single-stage procedure or as a two-stage procedure. The choice of the technique is multifactorial, depending on the characteristics of the diseased arch with regard to position of the stent graft proximal landing zone, patient fitness and comorbid status, as well as surgical expertise and hospital facilities. Among the evolving hybrid procedures is the so-called “frozen” or stented elephant trunk technique. Adapted from the classical elephant trunk technique, this approach facilitates the repair of a concomitant aortic arch and proximal descending aortic aneurysms in a single stage under circulatory arrest. This technique is increasingly being used to treat extensive thoracic aortic disease and has shown promising results.

## 1. Introduction

Open surgical repair of the aortic arch is a complex procedure, requiring cardiopulmonary bypass, hypothermic circulatory arrest and supra-aortic vessels reconstruction. Despite good results reported by centers performing a high volume of this procedure, open arch repair still presents a significant morbidity and mortality [1]. Since its introduction in 1994, aortic stent grafting has become the standard of care in descending aortopathies [2]. However, the application of stent graft technology into the aortic arch has been limited by specific challenges which mainly arise from the variation in anatomical configuration of the aortic arch and great vessels in conjunction with high blood flow rates and considerable motion. All these factors taken together present a difficult environment for total endovascular arch repair. Moreover, the presence of atherosclerotic disease in the arch presents a significant stroke risk during endovascular manipulation, and the proximity of the aortic valve and coronary arteries pose particular problems in more proximal pathology. For these reasons, hybrid repair that involves a combination of open surgery with endovascular aortic stent graft placement has been introduced as a therapeutic alternative for those patients deemed unfit for open surgical procedures [3]. Hybrid repair can be performed as a single-stage procedure or as a two-stage procedure, where the open component is performed first and then the endovascular component at a later point. Hybrid repair requires varying degrees of invasiveness. The choice of the technique is multifactorial, depending on the characteristics of the diseased arch with regard to position of the stent graft proximal landing zone, patient fitness and comorbid status, as well as surgical expertise and hospital facilities. Classification of hybrid approaches have been proposed by Bavaria and colleagues, according to the extent of aortic lesion and the presence of the proximal and distal landing zone [4].

(1) Type I Hybrid Repair involves supra-aortic debranching with subsequent endovascular repair of the aortic arch.
(2) Type II Hybrid Repair involves an open reconstruction of the ascending aorta and revascularization of the three branching vessels to create a proximal landing zone for an endovascular graft, which is then deployed to exclude the aneurysm.
(3) Type III Hybrid Repair consists of frozen elephant trunk procedure with surgical reconstruction of the aortic arch and revascularization of the branching vessels of the aortic arch. This procedure is reserved for patients with extensive aortic lesions involving the ascending aorta, transverse arch and the descending thoracic aorta.

In this review we will discuss surgical concepts and results of these techniques.

## 2. Type I Hybrid Repair

### 2.1. Surgical Techniques

The aim of the procedures is the creation of an adequate proximal landing zone for subsequent safe stent graft deployment. This is obtained with a supra-aortic vessels debranching (SaVD) by means of relocation of the arch’s branches with vascular grafts that can be routed through an anatomical or extra-anatomical path and they can be either intra or extra-thoracic. The SaVD is referred to the last aortic zone that has been excluded and follow the Ishimaru classification of zones of the aortic arch [5]: Zone 0 involves the proximal ascending aorta to the brachio-chepalic trunk (BCT) origin; Zone 1 is distal to the BCT but proximal to the left common carotid artery (LCCA); Zone 2 is distal to the LCCA but proximal to the left subclavian artery (LSA).

### 2.2. Zone 0 Landing Zone: Complete Arch Debranching

**Figure 1:**
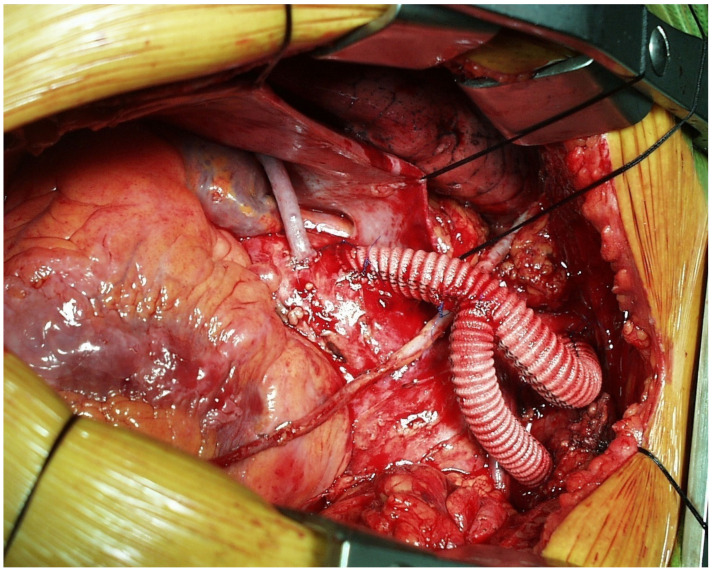
Zone zero aortic arch debranching with brachio-chepalic trunk (BCT) and left common carotid artery (LCCA) revascularization and concomitant coronary artery bypass grafting.

**Figure 2:**
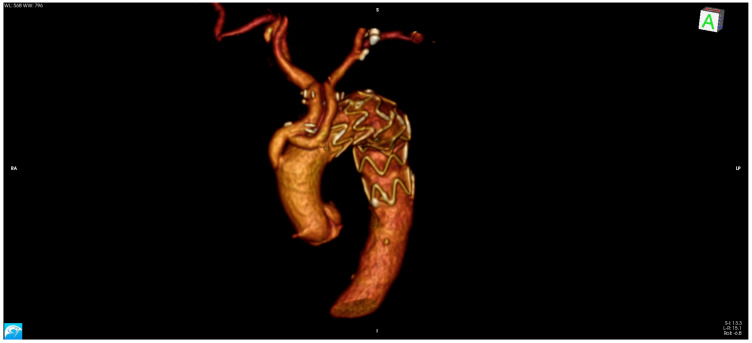
Three-dimensional (3D) angio-CT reconstruction of Zone 0 Type I Hybrid repair.

This approach is reserved for cases with isolated aortic arch aneurysm that have adequate proximal landing zone in the ascending aorta and distal landing zone in the descending thoracic aorta. The need to deploy the stent graft into the ascending aorta requires mandatory revascularization of the BCT and LCCA. The procedure is performed through a complete or partial median sternotomy. After mobilization of the supra-aortic vessels, the ascending aorta is tangentially clamped and an end-to-side anastomosis between the main trunk of a tri- or bifurcated dacron graft is performed. Due to extensive aortic manipulation, pharmacologically induced hypotension (target systolic arterial blood pressure ≤90 mmHg) is mandatory during this stage of the procedure. Finally, arch’s vessel reconstruction is performed from the LSA to the BCT. Each branch of the trifurcated vascular graft is trimmed and anastomosed to the corresponding native vessel. The LSA and LCCA are anastomosed in an end-to-end fashion, while the BCT in an end-to-side fashion. In effort to avoid type II endoleak is mandatory to ensure optimal occlusion of the proximal supra-aortic vessels stump. When the procedure is completed, endograft deployment can be performed concomitantly or at a later time. (Figure 1 and Figure 2).

### 2.3. Zone 1: Left Subclavian Artery (LSA) and Left Common Carotid Artery (LCCA) Debranching

This technique is usually performed when the aortic pathologies involve the mid and distal part of the aortic arch and the stent graft can be released between the BCT and the LCCA. The first surgical steps consist in LCCA and LSA revascularization with subsequent stent graft deployment immediately distal to the origin of the BCT. Usually this is accomplished with an extra anatomic-placed dacron vascular graft between the right and left common carotid arteries associated with a LCCA to LSA bypass. The procedure is performed with a bilateral neck incision. After bilateral common carotid arteries dissection, heparin is given and the RCCA is clamped. An end-to-side anastomosis with the dacron graft is then performed. The graft is then tunneled (anteriorly or in a retro-esophageal fashion), the LCCA is ligated, transected and a side to end anastomosis is performed. Finally the graft is anastomosed end-to-side with LSA. 

### 2.4. Zone 2: Covering of the LSA

This procedure is usually performed in patients with pathology of the distal arch or the proximal descending aorta who necessitate coverage of the LSA for adequate TEVAR implantation. Management of the LSA is still a matter of debate. Despite the fact that deliberate occlusion of the LSA can be performed safely in the majority of patients, some authors have suggested that revascularization of the LSA can reduce the incidence of post-procedure stroke [6,7]. The rationale for this is that coverage of the LSA may cause posterior circulation strokes by vertebrobasilar ischemia and anterior circulation strokes by indirectly reducing Circle of Willis perfusion pressures. Our indications for LSA revascularization include a patent left internal mammary artery for coronary artery bypass graft; small, occluded or absent right vertebral artery; dominant left vertebral artery; left arm arterio-venous fistula or patent left axillo-femoral bypass graft. 

LSA revascularization is obtained by means of a LCCA-LSA bypass or transposition. LCCA-LSA bypass is performed with a single supraclavicular neck incision. The LCCA and the postscalenic portion of the LSA are fully mobilized and encircled. After heparinization, an 8 mm dacron graft is anastomosed end-to-side with the LCCA. Then, a tunnel is created underneath the sternocleidomastoid muscle and the graft is anastomosed in end to side fashion with the subclavian artery. Usually, a vascular plug is placed at the origin of the subclavian artery in effort to avoid potential type II endoleak from the vertebral artery. 

An alternative to an LCCA-LSA bypass is represented by transposition of the subclavian artery to the LCCA. This procedure requires a more extensive dissection of the LSA with partial or total anterior scalene muscle division. After the vertebral artery and internal mammary artery are isolated, the proximal stuff of the LSA is ligated and divided. Then, the LSA is anastomosed in end-to-side fashion with the LCCA. The advantage of this procedure is the avoidance of any prosthetic material. However the technique is more complex than the LCCA-LSA bypass. 

## 3. Type II Hybrid Repair

### Surgical Technique

**Figure 3:**
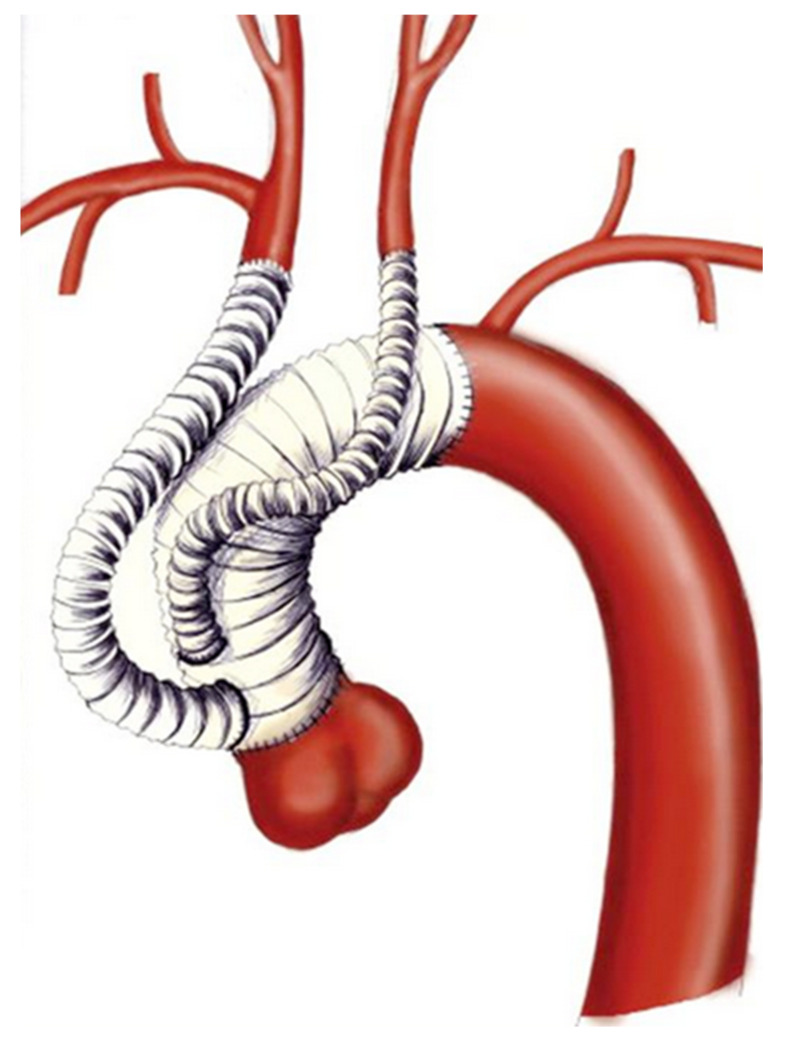
Schematic illustration of Type II Hybrid repair.

**Figure 4:**
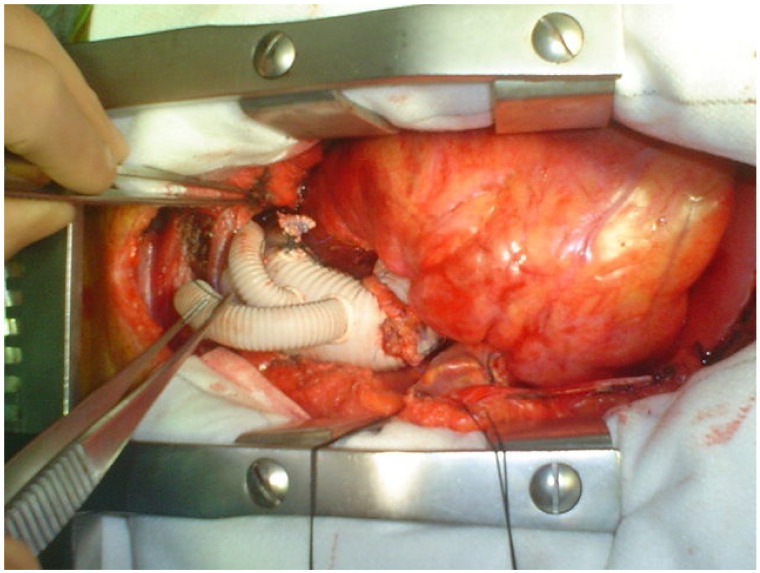
Type II Hybrid Repair with a dedicated branched vascular prosthesis (Plexus (Neenah, WI, USA), Vascuteck (Inchinnan, UK)).

Type II Hybrid repair is indicated in those patients who do not have a suitable ascending aorta, such as an aortic arch aneurysm that extends proximally into the ascending aorta. The technique involves an open repair of the ascending aorta and revascularization of the three branching vessels to create a proximal landing zone for an endovascular graft which is then deployed to exclude the aneurysm. Type II hybrid repair allows a prosthetic proximal landing zone to be created which guarantees mechanical stability and caliber uniformity, thus resulting in a more homogenous sealing and in a less probability of developing endoleak. In addition, the prosthetic landing zone is potentially less susceptible to aortic remodeling than the native aorta and, therefore, may be more resistant to delayed endoleak. The procedure is performed through a partial or complete sternotomy with CPB and cardioplegic arrest. After ascending aorta replacement, SAV debranching is performed as previously described or with the use of a trifurcated vascular graft. In those patients with associated proximal arch pathology, partial arch replacement with circulatory arrest and antegrade selective cerebral perfusion is indicated. (Figure 3 and Figure 4).

## 4. Results of Type I and Type II Hybrid Repair

Current evidence of hybrid procedures is mostly limited to observational studies with short-term follow-up. Despite progressive advancement of surgical techniques and stent graft technology, hybrid arch repair results have not changed significantly over the years. In 2010 Koullias et al., published results from a meta-analysis on 15 studies reporting a pooled overall 30-day mortality of 8.3%, endoleak rate of 9.2%, stroke of 4.4%, and paraplegia of 3.9% [8]. Antoniou et al. conducted a meta-analysis of hybrid supra-aortic debranching and stent graft repair [9]. A pooled analysis of 275 patients showed a mean 30-day mortality rate of 15%, stroke rate of 8% and paraplegia rate of 2%. Similar results have been reported in 2013 by Moulakakis and colleagues: 30-day mortality of 11.9%, endoleak rate of 16.6%, stroke rate of 7.6%, and spinal cord injury rate of 3.6% [10].

A meta-analysis of seven retrospective cohort studies evaluating hybrid arch repair versus open surgical repair of an aortic arch aneurysm reported no significant difference in relation to neurological complications, late mortality and renal failure, between the two techniques. However open surgical repair was associated with a significantly lower rate of re-intervention [10]. Similar results have been reported by Miao and Tokuda [11,12]. Furthermore, in a meta-analysis looking at four observational studies comparing open repair with a hybrid technique in a total of 378 participants, Di Benedetto and colleagues found that hybrid repair was not associated with reduction in surgical deaths but was associated with a slight increase in permanent neurologic deficit [13]. 

Long-term results of Hybrid Arch Repair are not well established. Complications at the level of the proximal landing zone, such as endoleaks or retrograde aortic dissection, represent the primary failures of hybrid procedures. Type I or attachment-site endoleak (persistent blood flow outside the lumen of the stent graft within the aneurysm sac), lead to progressive aneurysmal dilatation and frequently require conversion to open surgery and close follow-up [14,15]. Also, Retrograde type A aortic dissection is a serious complication that requires emergency surgery and is associated with an high operative mortality and morbidity [16]. Predictive characteristics that may represent risk factors for proximal landing zone complications are: a short proximal landing zone, angulation of the aortic arch, incorrect sizing and procedural mistakes. This raises the issue of the ideal proximal landing zone, whose quality and stability largely determines the technical success of the procedure. The ideal parameters for landing zones in the aortic arch include an aortic diameter smaller than 37 mm, a length of disease-free aorta of at least 20 mm, and an angulation of less than 60 degrees. Moreover, the proximal landing zone must be free of calcium and mural thrombus [17,18]. 

Another aspect of the hybrid technique is the long-term patency of bypass grafts. Occlusion or stenosis of a supra-aortic bypass graft represent a serious complication that can lead to upper limb ischemia, stroke and death. Despite this, patency rates are often under-reported in studies involving the hybrid technique so little is known about their long-term durability. 

## 5. Type III Hybrid Repair: The Frozen Elephant Trunk (FET)

Frozen Elephant Trunk (FET) is an elaborate procedure which aims to simplify the treatment of complex thoracic aortic lesions. The technique consists in antegrade delivery of a stent graft into the open proximal descending thoracic aorta during hypothermic circulatory arrest followed by reconstruction of the aortic arch and ascending aorta. Indications for FET should be based on both the underlying aortic pathology and the patient’s characteristics [19]. Since its development in 2003, FET has been extensively used in patients with thoracic or thoracoabdominal aortic pathology (degenerative or post dissection) when a second procedure can be anticipated. In this subset of patients, the FET allows an extensive primary repair of aortic arch and descending aorta and facilitates any secondary future intervention on the distal thoracic aorta reducing the risk of additionally distal aortic surgery.

In the last decade, the use of FET has been extended also to patients with type A acute aortic dissection. The rationale lies in the potential for prevention of late distal aortic complications by total thoracic aortic remodeling, which may result from stenting the descending thoracic aorta during the acute phase of aortic dissection. Indeed, unsatisfactory long-term prognosis including aneurysmal degeneration, rupture, malperfusion, and the need for extensive secondary or tertiary interventions has been reported in a large percentage of survivors after conventional type A acute dissection repair [20]. Also patients with complicated acute type B aortic dissection when primary TEVAR is not feasible represent an indication for FET [21].

Nowadays, two commercially available hybrid prostheses are available in Europe for FET interventions: the E-vita OPEN NEO hybrid stent graft system (JOTEC CryoLife, Kennesaw, GA, USA) and the Thoraflex™ Hybrid (Vascutek, Inchinnan, UK).

The E-vita OPEN NEO hybrid graft is available with the dacron arch graft shaped in three different variants: (1) a straight graft with a side branch for lower body perfusion, (2) a graft having individual branch grafts for selective anastomosis to the supra-aortic arch vessels, (3) a graft with a single large trifurcated branch for arch vessel reconstruction specifically designed for Zone 0 reconstruction. The length of the stent-graft varies from 120 to 180 mm and the diameters from 22 to 40 mm. 

The Thoraflex™ Hybrid graft is composed of a stent graft and a four-branched dacron graft for individual arch vessel reimplantation and lower body perfusion. The stent graft is available in diameters of 28–40 mm (for the stented portion) and the length of the stent graft is either 100 or 150 mm.

**Figure 5:**
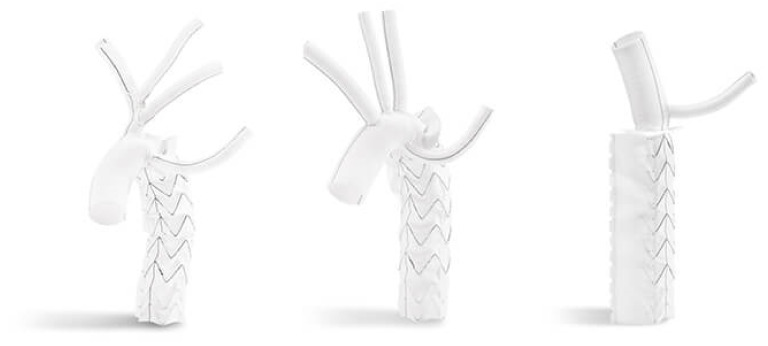
The Cryolife Jotec E-vita OPEN NEO hybrid stent graft system is composed by a proximal dacron prosthesis for the arch and a distal self-expandable stent graft with Z-nitinol stents for the descending thoracic aorta.

**Figure 6:**
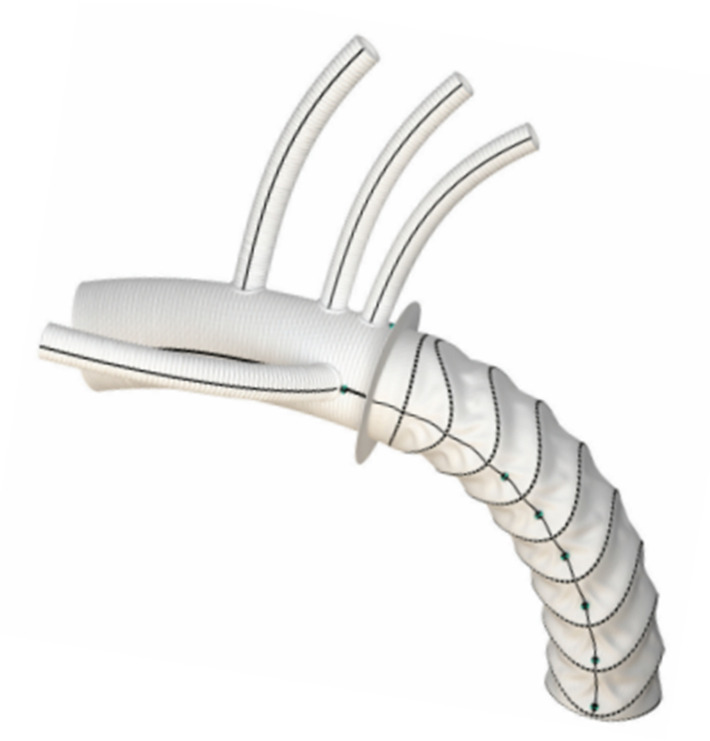
Vascutek Thoraflex hybrid device is composed of a proximal four-branched pre coated woven polyester prosthesis sealed to a distal covered stent graft. The stented portion of the graft is a self-expanding endoprosthesis made with polyester and nitinol ring stents, which are attached to a fabric with braided polyester sutures.

They both have a sewing collar to facilitate distal anastomosis.(Figure 5 and Figure 6).

### 5.1. Surgical Technique

The procedure is performed through a median sternotomy. Cardiopulmonary bypass is established and the patient is cooled to 28 °C. Femoral artery, right axillary artery, the brachiocephalic trunk or ascending aorta can be cannulated for arterial inflow, depending on surgeon’s preference and patient’s characteristics. During systemic cooling, the LSA is anastomosed with an 8 mm dacron graft and LSA perfusion is initiated. When target core temperature is reached, the aorta is clamped and the heart is arrested. Consecutively, under hypothermic circulatory arrest the aortic arch is opened, the BCT and LCCA are cannulated and antegrade selective cerebral perfusion (ASCP) is initiated. Our protocol for ASCP involves a flow rate of 10 mL/kg/min adjusted to maintain a right radial artery pressure between 60 and 80 mmHg. Resection of the aortic arch is extended until the target zone for anastomosis and the aortic wall is then reinforced with an extraluminal strip of Teflon. The stented portion of the FET device is then advanced in the thoracic aorta and deployed over a guide wire introduced from the femoral artery. The device is then anastomosed to the aortic wall with a running suture and systemic reperfusion with rewarming is established through a side branch of the prosthesis. Consecutively the proximal anastomosis between the graft and the ascending aorta is performed. Finally, the supra aortic vessels reconstruction is obtained from the LSA to the BCT, using the branches of the trifurcated graft.

### 5.2. Results of the FET

In recent years, FET has been increasingly used in most centers worldwide both for chronic and acute aortic pathologies. When used by experienced surgeons on selected patients, these techniques do not seem to be associated with an increased risk of hospital death or stroke [22]. In a recent review by Tian and colleagues on 4172 pooled patients, the authors reported mortality, permanent neurological deficit and spinal cord injury rates of 10.2%, 7.7%, and 6.5%, respectively [23]. In addition overall survival at 5 years was 82.0% and freedom from re-intervention at the same time point was 86.8%. In the setting of Type A acute aortic dissection, Di Eusanio and colleagues reported early mortality of 10% (range 0–27.7%) with a postoperative stroke rate of 4.8% (range 0–12%) [24].

Another advantage of FET is the potential for aortic remodeling. The International E-Vita Open Registry reported that FET promotes false lumen thrombosis, and remodeling of the descending thoracic aorta in 99.3% of patients. However increased false lumen thrombosis and positive remodeling rates were not maintained at the level of the abdominal aorta (13.9%) [25]. Similar results of promising rates of false lumen partial/complete thrombosis have been reported by other authors suggesting that aortic remodeling is highly probable with this approach. [24,26].

One of the main drawbacks of FET is its potential association with spinal cord ischemia. The pathophysiology of spinal cord injuries is multifactorial and is associated with the length of coverage of the intercostal arteries, incomplete revascularization of the left subclavian artery and prolonged systemic hypotension. Preventza et al., reported results of a meta-analysis of more than 3000 patients who underwent FET. The authors found a pooled incidence of spinal cord injury of 4.7% and a significant association of spinal cord injury with the use of long stents (≥15 cm) and extension of coverage to T8 [27].

## 6. Conclusions

In summary, aortic arch aneurysms pose a formidable surgical challenge. Open repair requires cardiopulmonary and hypothermic circulatory arrest, but is safe and represents the gold standard for surgical repair. Hybrid techniques have shown promising results; however, hybrid arch repair presents high rates of neurologic events and carries higher rates of re-intervention when compared with open surgical repairs. For these reasons, hybrid techniques should be reserved for those patients who have a clear contraindication to surgery. FET is a complex procedure that has significantly simplified treatment of extensive aortic pathologies. Early and long-term results are encouraging and with the development of new devices that facilitate FET implantation, this procedure would extend the indications and gain popularity.
